# Elucidating the mechanism of action of alpha-1-antitrypsin using retinal pigment epithelium cells exposed to high glucose. Potential use in diabetic retinopathy

**DOI:** 10.1371/journal.pone.0228895

**Published:** 2020-02-07

**Authors:** María Constanza Potilinski, Gustavo A. Ortíz, Juan P. Salica, Emiliano S. López, Mariano Fernández Acquier, Eduardo Chuluyan, Juan E. Gallo

**Affiliations:** 1 Nanomedicine & Vision Group, Facultad de Ciencias Biomédicas, Instituto de Investigaciones en Medicina Traslacional, Universidad Austral, Consejo Nacional de Investigaciones en Ciencia y Tecnología (CONICET), Pilar, Buenos Aires, Argentina; 2 Department of Ophthalmology, Hospital Universitario Austral, Pilar, Buenos Aires, Argentina; 3 Servicio de Neumonología, Hospital Cetrángolo, Vicente López, Buenos Aires, Argentina; 4 Centro de Estudios Farmacológicos y Botánicos, CONICET (CEFYBO), Facultad de Medicina, Universidad de Buenos Aires, Buenos Aires, Argentina; University of Florida, UNITED STATES

## Abstract

**Background:**

Alpha-1-antitrypsin is a protein involved in avoidance of different processes that are seen in diabetic retinopathy pathogenesis. These processes include apoptosis, extracellular matrix remodeling and damage of vessel walls and capillaries. Furthermore, because of its anti-inflammatory effects, alpha-1-antitrypsin has been proposed as a possible therapeutic approach for diabetic retinopathy. Our group tested alpha-1-antitrypsin in a type 1 diabetes mouse model and observed a reduction of inflammation and retinal neurodegeneration. Thus, shedding light on the mechanism of action of alpha-1-antitrypsin at molecular level may explain how it works in the diabetic retinopathy context and show its potential for use in other retinal diseases.

**Methods:**

In this work, we evaluated alpha-1-antitrypsin in an ARPE-19 human cell line exposed to high glucose. We explored the expression of different mediators on signaling pathways related to pro-inflammatory cytokines production, glucose metabolism, epithelial-mesenchymal transition and other proteins involved in the normal function of retinal pigment epithelium by RT-qPCR and Western Blot.

**Results:**

We obtained different expression patterns for evaluated mediators altered with high glucose exposure and corrected with the use of alpha-1-antitrypsin.

**Conclusions:**

The expression profile obtained *in vitro* for the evaluated proteins and mRNA allowed us to explain our previous results obtained on mouse models and to hypothesize how alpha-1-antitrypsin hinder diabetic retinopathy progression on a complex network between different signaling pathways.

**General significance:**

This network helps to understand the way alpha-1-antitrypsin works in diabetic retinopathy and its scope of action.

## 1. Introduction

Diabetic retinopathy (DR) is the principal cause of visual loss and blindness in the working age population. Among diabetics the estimated global prevalence of DR is 35.4% [1]. The leading contributor to the development of DR is hyperglycemia [2]. According to the National Eye Institute, DR is classified as non-proliferative or proliferative and is characterized by changes in the retina involving microaneurysms, hemorrhages, hard and cotton-wool exudates, edema, neovessels and, eventually, retinal detachment [3]. Current treatments available for DR, such as laser photocoagulation, intravitreal injections of anti-vascular endothelial growth factor (VEGF) molecules and corticosteroids, as well as vitreo-retinal surgery, are non-preventive and applicable on advanced stages of the disease [4]. Considering that the number of people affected by diabetes mellitus has increased from 5% to 10% in the last 25 years and continues growing [5], and taking into account that DR is the most common secondary complication, new approaches are needed for the treatment or prevention of DR in earlier stages. Modern treatments could result in lower socioeconomic costs for health care systems and improved life quality for diabetics.

### 1.1 Retinal pigment epithelium & diabetic retinopathy

The retinal pigment epithelium (RPE) is a single layer of epithelial cells located on Bruch’s membrane between the choroid and neural retina. The RPE performs different functions, including turnover of photoreceptor outer segments and oxidative stress response, and plays an important role in allowing phototransduction [6]. The RPE forms the outer blood-retinal barrier where adhesion and communication between RPE cells is essential to prevent the passage of molecules and ions, and maintain cell polarity. These events allow the correct functioning of the retina and maintain retinal immune privilege [6,7]. Most researchers agree that one of the early events in DR is the dysfunction of RPE, affecting the retina [8]. In DR, RPE dysfunction is consequence of hyperglycemia, leading to a dysregulation on different protein expression, which, in turn, contributes to oxidative stress and, eventually, angiogenesis [9,10].

### 1.2 Alpha-1-antitrypsin

Alpha-1antitrypsin (A1AT) is a sialoglycoprotein of 52kDa, encoded by the gene SERPINA1 [11]. It is produced as an acute phase protein by hepatocytes in the liver, but it is also produced in less amounts in intestinal epithelial cells, lungs, neutrophils and alveolar macrophages [12]. A1AT commonly works as a protease inhibitor of proteins like neutrophil proteases related to inflammation processes, such as proteinase-1, elastase, thrombin and trypsin [12,13]. Serum concentrations of A1AT change during a disease or in response to inflammation or tissue injury [14]. A1AT is currently used to treat chronic obstructive pulmonary disease and A1AT deficiency [15,16]. Recently, A1AT has been proposed as a possible therapeutic approach for diabetic retinopathy based on its anti-inflammatory effects. In fact, A1AT is a molecule involved in several mechanisms observed in DR, such as anti-inflammatory processes, avoidance of apoptosis and extracellular matrix remodeling, as well as protection of vessel walls and capillaries [17]. Furthermore, our group tested A1AT in a type 1 diabetes mouse model (streptozotocin model) and observed a reduction of inflammation and retinal neurodegeneration. Systemic treatment of A1AT downregulated NFkB, iNOS and TNF-alpha expression. These factors showed a positive correlation with retinal degeneration, highlighting their key role in DR pathogenesis [18]. Consequently, more information about the mechanism of action of A1AT is needed to learn both how its administration ameliorates cytokine levels (like reducing TNF-α) and its scope of action on cell processes in the retina. In addition, new insights on A1AT could be useful for other retinal diseases.

In this work, we intended to evaluate the use of A1AT in ARPE-19 cells exposed to high glucose and explore the NFκB pathway involved in the production of TNF-α and iNOS pro-inflammatory cytokines, the PI3K/Akt pathway that plays an important role in several cellular processes such as glucose metabolism, transcription, apoptosis and cell migration, as well as the Wnt pathway that takes part in cell proliferation, tissue regeneration and cell migration. Based on this, we also included the study of molecules related to the previous mentioned pathways. This allowed us to identify core elements that connect different pathways and show how A1AT plays a role in different processes related to DR and normal function of RPE cells.

## 2. Materials and methods

### 2.1 ARPE-19 cell culture

ARPE-19 cells exposed to high glucose (Retinal Pigment Epithelial immortalized cell line derived from Amy Aotaki-Keen eyes) is a widely used *in vitro* model of diabetic retinopathy. Exposing cells to high glucose concentrations (from 16.5 mM to 33 mM) with 10% fetal bovine serum mimics diabetic retinopathy milieu [19–23].

ARPE-19 cells (ATCC^®^ CRL-2302TM, Manassas, Virginia, USA) were cultured in Dulbecco’s modified Eagle’s medium/nutrient mixture F12, DMEM/F12 (Gibco, Invitrogen Corporation, Carlsbad, California, USA) containing: 2 μM L-glutamine, 100 U/ml penicillin, 100 μg/ml streptomycin, and 10% fetal bovine serum (Natocor, Córdoba, Argentina) and maintained at 37°C and 5% CO_2_. ARPE-19 cells were used from passages 9 to 12 and cultured at a seeding density of 1 × 10^6^ cell. After reaching 90% of confluence, cells were incubated in Dulbecco’s modified Eagle’s medium/nutrient DMEM with low glucose (Gibco, Invitrogen Corporation, Carlsbad, California, USA) for 16 hours supplemented with A1AT (Prolastin^©^C, Grifols, Spain) 4.5 mg/ml and/or 30 mM glucose. Four groups were made and divided into: control (DMEM low glucose, 5.5 mM), control + A1AT (DMEM 5.5 mM glucose, 4.5 mg/ml A1AT), high glucose (DMEM 30 mM glucose), high glucose + A1AT (DMEM 30 mM glucose, 4.5 mg/ml A1AT).

### 2.2 MTT assay

Cell viability was evaluated by MTT assay. Cells were seeded in a 96 well plate (10000/well) and incubated with the corresponding treatments overnight. Cells were incubated with MTT (Thiazolyl Blue Tetrazolium Bromide) reagent (Sigma-Aldrich) for 4 hours at 37°C. On viable cells MTT is converted to dark blue, water-insoluble MTT formazan by mitochondrial dehydrogenases. Precipitate was solubilized in acidified isopropanol and measured by spectrophotometry at 570 nm. Each sample was seeded by quintuplicate and five assays were performed.

### 2.3 Acridine orange/ethidium bromide

Cells were seeded on cover glasses and incubated with different supplemented media according to experimental groups. After treatment, cells were washed with ice cold PBS buffer, exposed to 10μl of the dual fluorescent staining solution including 100 μg/ml acridine orange (AO) and 100 μg/ml ethidium bromide (EB) (AO/EB, Sigma, St. Louis, MO) and covered with a coverslip. Apoptotic cells were scored (500 cells per slide) and their morphology was examined. Images were taken with Nikon Eclipse Ni-E, DS-Ri2 camera, total magnification 400X (10X ocular lenses and 40X objective), NIS Elements camera software. Triplicates were obtained for each experimental point.

### 2.4 RT-qPCR

ARPE-19 cells were harvested in Trizol Reagent (Sigma-Aldrich) to extract total RNA according to manufacture proceedings. 1 μg of total RNA was reverse transcribed with 200 Units of Superscript II Reverse Transcriptase (Invitrogen, Carlsbad, California, USA) using 500 ng of Oligo (dT) primers. cDNAs obtained were subjected to real-time polymerase chain reaction (qPCR) using the following primers (Table 1):

**Table 1:** Primers used in qPCR to amplify mRNA corresponding to genes of interest in this work. All the sequences are expressed in sense 5’-3’.

Protein	Gene	Forward 5'-3'	Reverse 5'-3'
Cx43	GJA1	TTTCATTAGGGGGAAGGCGTGAGG	TCCCTCCAGCAGTTGAGTAGGCT
Actin	ACTB	CAATCAGCGTGCGCCGTTCC	GGCCTCGTCGCCCACATAGG
NKAα1	ATP1A1	GGTTCCTGCCATCTCCCTGGC	CCACTCGGAGGCCCAACAGG
NKAα2	ATP2A2	TCGACTGGGATGACCGGACCA	GAAAGGCAGCCAACGCCGTC
NKAα3	ATP3A3	GTTGTCGTCCAGTGGGCCGAT	GCCAGGGCCGTCTCCTCAAA
Axin2	AXIN2	CCACACCCTTCTCCAATCCAAGCC	ACCGCTCCACAGGCAAACTCATC
Bcl2	BCL2	GACGCTTTGCCACGGTGGTG	GCATGCTGGGGCCGTACAGT
N-Cadherin	CDH2	GGATGAAACGCCGGGATAAAGA	TTCATCCATTCGTCGGATTCCC
β-Catenin	CTNNB1	ACGGCTTTCAGTTGAGCTGACCA	CCAAGGGGTTCTCCCTGGGC
FOXO1	FOXO1	AGGGTGACAGCAACAGCTCGG	GCTCGGCTTCGGCTCTTAGCA
GSK-3β	GSK3B	TGGGCGAGACACACCTGCAC	GCATCTGACGCTGCTGTGGC
IRS-1	IRS1	GCAGCCAGAGGACCGTCAGT	TGACACGGTGGTGGGCACAT
mTOR	MTOR	TGCCGTCCAGGGCTTCTTCC	CCACCAAGGGTCTGGGCGTA
PTEN	PTEN	GGCGGAACTTGCAATCCTCAGTTT	TCTGAGGTTTCCTCTGGTCCTGGT
AS160	TBC1D4	AGGAGTTCCCAAAAGTCGACGAGG	GCTGTCCTGGCCCAAGCTGT
TNF-α	TNFA	GTGATCGGCCCCCAGAGGGA	CGGCGGTTCAGCCACTGGAG

qPCR amplifications were carried out using a cycle of 95°C for 10 minutes and 40 cycles under the following parameters: 95°C for 30 seconds, 60°C for 1 minute, 72°C for 30 seconds (Stratagene Mx3005p, Stratagene, La Jolla, California) with SYBR Green PCR Master Mix (4309155, Applied Biosystems). At the end of the PCR, the temperature was increased from 60 to 95°C at a rate of 2°C/minute, and fluorescence was measured every 15 seconds to construct the melting curve. Values were normalized to levels of ACTB (β-Actin, housekeeping). Data were processed by the ΔΔCt method [24]. The relative amount of the PCR product amplified from the control group was set as 1. A non-template control was run in every assay and all determinations were performed as triplicates for each sample in three separate experiments.

### 2.5 Western blot

ARPE-19 cells were harvested in RIPA buffer supplemented with protease inhibitor cocktail (Sigma-Aldrich P2714) and Sodium orthovanadate 1 mM. Proteins were quantified by Bradford assay and 20 μg of each sample was loaded and analyzed by SDS-PAGE on 15%, 10% or 8% SDS-Polyacrylamide gel, as needed, followed by electrophoresis and electroblotting onto polyvinylidene difluoride membranes (PVDF; Amersham, Hybond-P RPN303F, GE Healthcare, Chicago, Illinois, USA) by wet transfer. Membranes were incubated overnight at 4°C with antibodies against goat anti-Actin (1/700, C11 sc-1615, Santa Cruz Biotechnology), rabbit anti-Akt (1/1000, 4691, Cell Signaling), rabbit anti-pAkt 1/2/3 Ser473 (1/200, sc-7985-R, Santa Cruz Biotechnology), rabbit anti-Cx43 (1/300, H-150 sc-9059, Santa Cruz Biotechnology), rat anti-E-Cadherin (1/300, DECMA-1 sc-59778, Santa Cruz Biotechnology), rabbit anti-FGFR1 (1/1000, 9740, Cell Signaling), rabbit anti-GLUT1 (1/1000, 129395, Cell Signaling), mouse anti-HIF-1α (1/300,28b sc-13515, Santa Cruz Biotechnology), rabbit anti-IRS1 (1/1000, ab652, Abcam), rabbit anti-NFκB p65 (1/1000, 8242, Cell Signaling), rabbit anti-TBC1D4 (1/1000, ab189890, Abcam) in this work AS160 is used as an alternative name for TBC1D4 because it refers as Akt substrate of 160 KDa, mouse anti-Transglutaminase2 (1/1000, ab2386, Abcam), rabbit anti-TNF-α (1/1000, ab6671, Abcam). After incubation with primary antibodies, membranes were incubated for 1.5 hours at room temperature with anti-goat-Horseradish peroxidase (HRP) (1/8000, A5420, Sigma Aldrich), anti-rabbit-HRP (1/50000, A0545, Sigma Aldrich), anti-mouse-HRP (1/3000, ab6728, Abcam), anti-rat-HRP (1/3000, ab6734, Abcam). Finally, membranes were incubated with ECL (Pierce, Thermo Scientific, 32132) and chemiluminescent signal was detected with high performance chemiluminescence film (Amersham, Hyperfilm ECL, GE Healthcare, Chicago, Illinois, USA). Immunoblot quantifications were performed with FIJI (doi:10.1038/nmeth.2019) and relativized to Actin.

### 2.6 Immunohistochemistry

Cells were seeded on cover glasses and incubated with different supplemented media according to experimental groups. After treatment, cells were washed with ice cold PBS buffer and fixed with cold 4% formaldehyde solution in PBS for 10 minutes. Fixed cells were incubated with the primary antibodies rabbit anti-NFκB (1/100, 82425, Cell Signaling) and rabbit anti-iNOS (1/100, ab3523, Abcam) overnight at 4°C. Primary antibodies were removed, and cells were washed with PBS and incubated with the secondary antibody anti-rabbit-FITC (1/50, sc-2012, Santa Cruz Biotechnology) at room temperature for 2 hours. After incubation with secondary antibodies, cells were washed with PBS and nuclei were stained with DAPI (4',6-Diamidino-2-Phenylindole, Dihydrochloride) before mounting with glycerol with Dabco^®^ (Sigma-Aldrich) antifading. Images were taken with Nikon Eclipse Ni-E, DS-Ri2 camera, total magnification 400X (10X ocular lenses and 40X objective), NIS Elements camera software.

### 2.7 Statistical analysis

Three independent experiments were done in this work. Measurements were expressed as mean ± SD and statistically analyzed with ANOVA and Tukey multiple comparison test with Prism 7 for Mac OS X, version 7.0a 2016 (GraphPad Software Inc, San Diego, California). A *p* value <0.05 was considered to be statistically significant.

## 3. Results

### 3.1 A1AT did not affect viability of ARPE-19 cells

**Fig 1:**
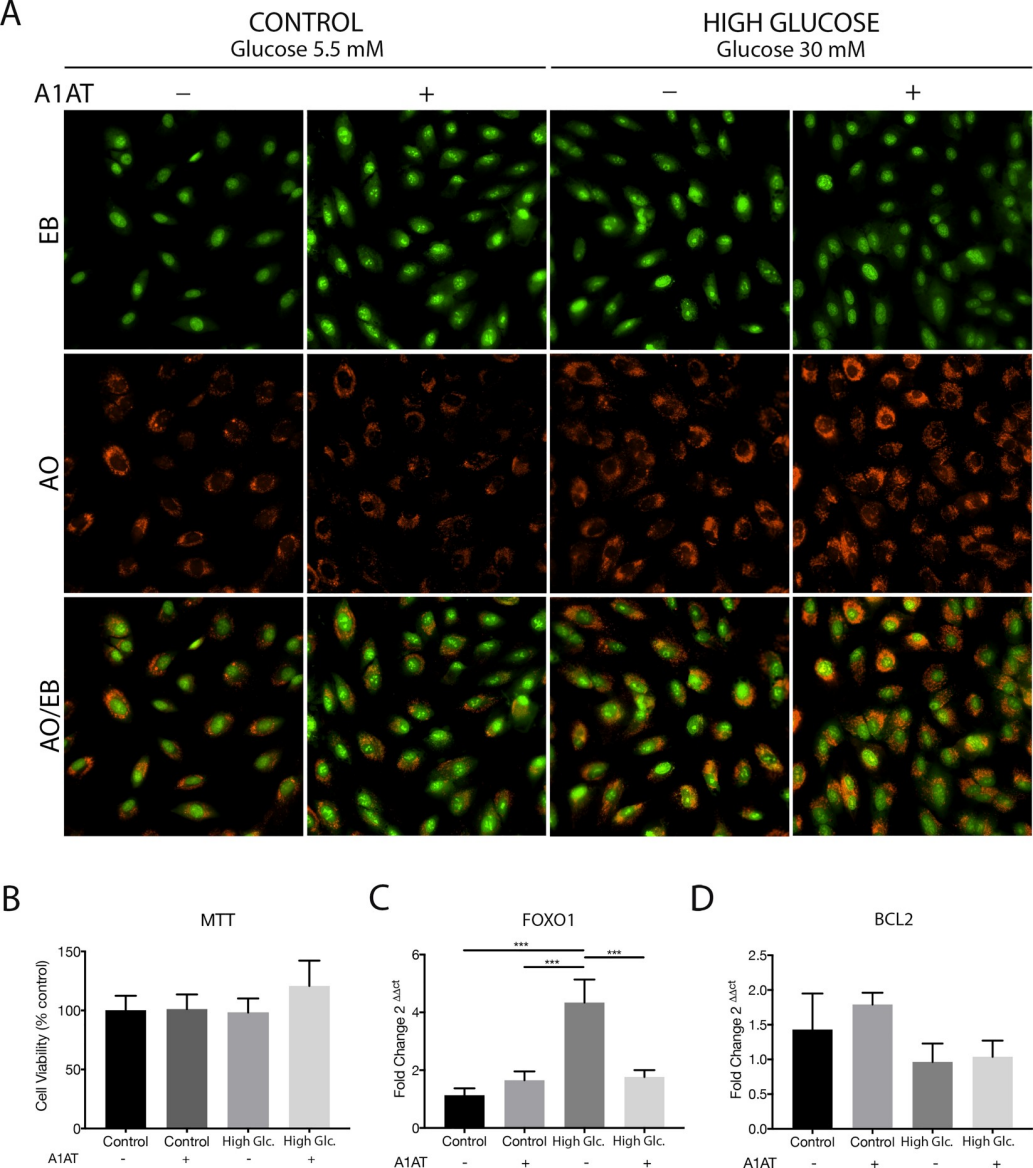
Viability of ARPE-19 cells treated with high glucose (High Glc.) and with A1AT. (A) Representative microphotographs of AO/EB staining assay in different cell culture conditions, total magnification 400X (10X ocular lenses and 40X objective). (B) Viability determined with MTT assay. Data expressed as percentage of control cultures, mean ± SD (n = 5). (C) FOXO1 and (D) Bcl2 mRNA expression levels measured with RT-qPCR. Data expressed as fold change with respect to control. ***p< 0.001 vs. Control.

ARPE-19 cells treated with high glucose concentration media (glucose 30 mM) and with A1AT (4.5 mg/ml) were evaluated by AO/EB and MTT assays to assess the viability of cells under culture conditions. No apoptotic cells were detected with AO/EB (Fig 1A) and no viability changes were observed with MTT assay (Fig 1B). Also, mRNA of FOXO1 and BCL2, two pro-apoptotic genes, was measured by RT-qPCR, and higher levels of FOXO1 were observed in the high glucose group (Fig 1C). No significant changes were observed with A1AT treatments or on BCL2 levels (Fig 1D).

### 3.2 A1AT decreased NFκB, and levels of its targets iNOS and TNF-α improved with high glucose

**Fig 2:**
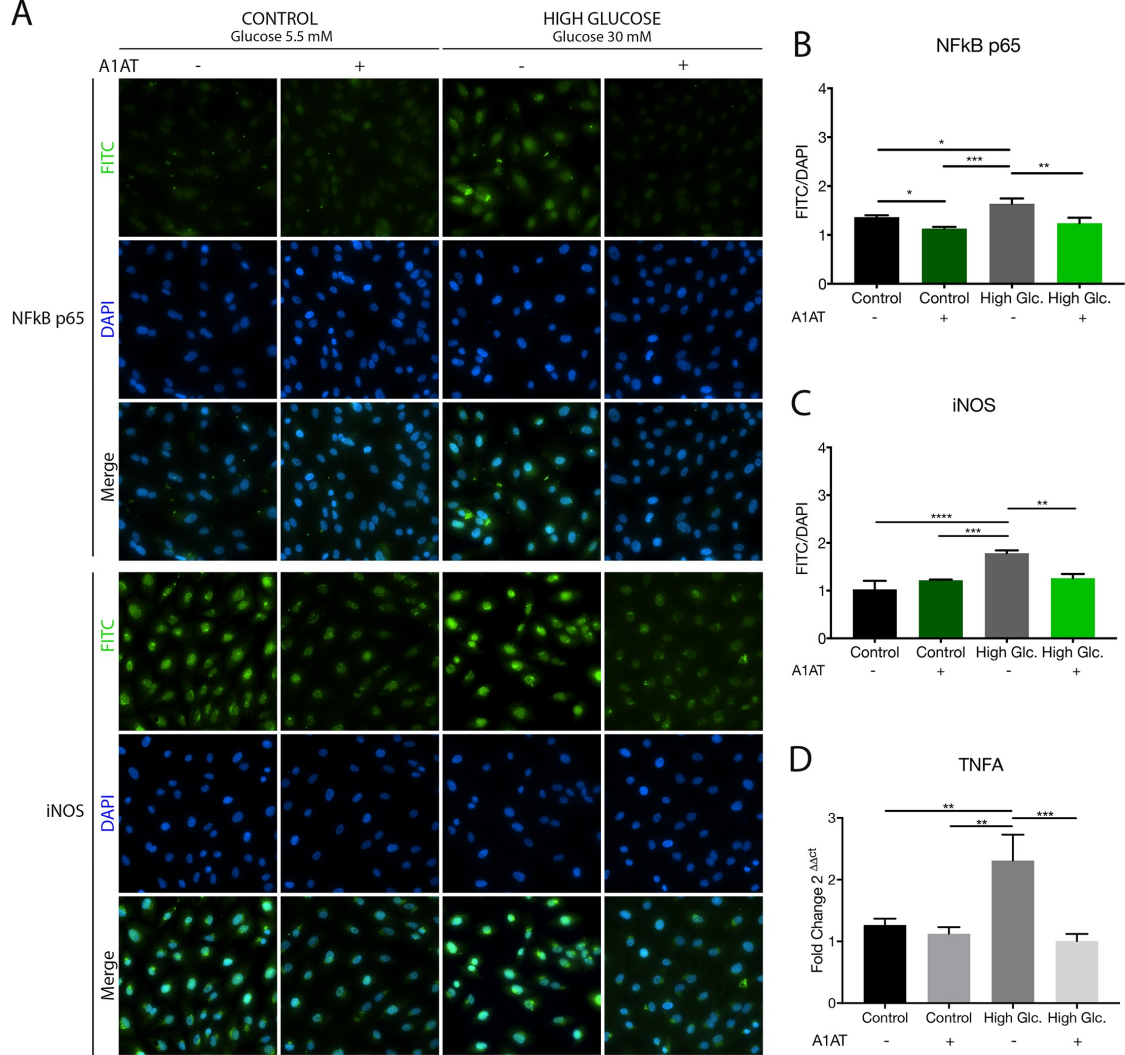
Reduced expression of NFκB and inflammation related targets iNOS and TNF-α on high glucose group treated with A1AT. (A) Representative microphotographs of NFκB p65 and iNOS immunohistochemistry and DAPI staining under control, high glucose and A1AT treatment culture conditions; total magnification 400X (10X ocular lenses and 40X objective). (B and C) Quantification of immunofluorescence assay, data expressed as mean of FITC fluorophore signal vs. DAPI signal ± SD (n = 5). (D) TNF-α mRNA expression levels measured by RT-qPCR. High glucose group expressed as ‘High Glc’. Data expressed as fold change with respect to control.*p< 0.05, **p< 0.01, ***p< 0.001 vs. Control.

**Fig 3:**
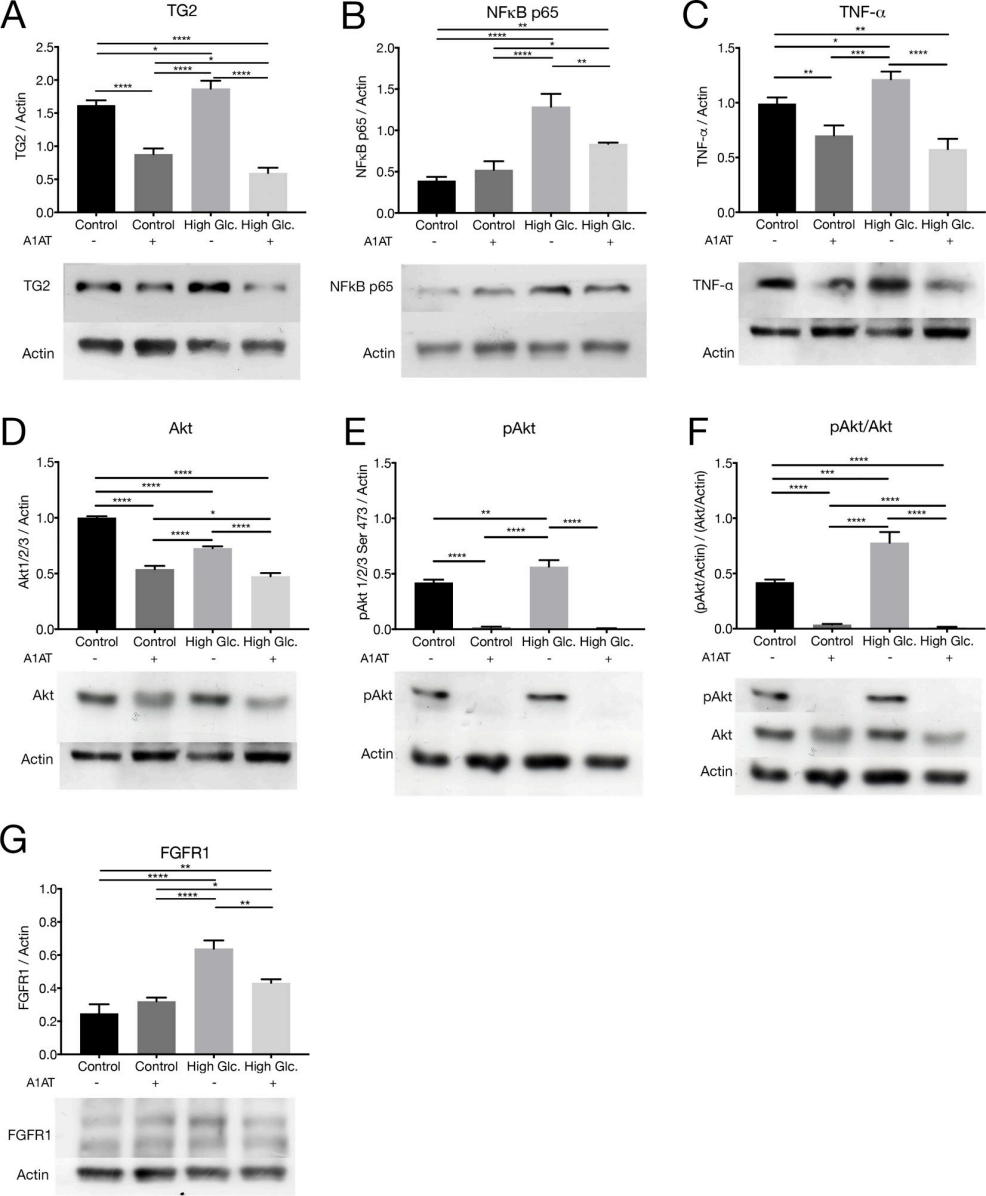
NFκB pathway under high glucose and with A1AT treatment. Expression of different intermediates of NFκB pathway was measured by western blot (A) Transglutaminase 2, (B) NFκB p65, (C) TNF-α, (D) Akt, (E) pAkt, (F) levels of pAkt relativized to Akt, (G) FGFR1. Results were expressed as protein vs. Actin expression mean ± SD. *p< 0.05, **p< 0.01, ***p< 0.001, ****p< 0.0001 vs. Control. Due to the different molecular weight of Akt, TNF-α and Actin, the same PVDF membranes were incubated with the different antibodies, for this reason, Actin band pattern is repeated between Akt and TNF-α. In pAkt/Akt ratio Akt Actin was choosen to display as an example, pAkt was relativized to its actin and the same procedure was done to Akt, then pAkt/Actin was relativized to Akt/Actin. The actin and Akt bands in (E) and (F) are almost identical.

NFκB p65 is a nuclear transcription factor and its targets iNOS and TNF-α are normally increased in diabetic models and related inflammation. Thus, to analyze if levels of NFkB, iNOS and TNF-α were affected by A1AT we performed a fluorescence immunohistochemistry of NFκB p65 and iNOS (Fig 2A, Fig 2B, Fig 2C). Also, a RT-qPCR for TNF-α mRNA was made (Fig 2D). In all cases an increased expression of each protein was observed under high glucose conditions and normalized with A1AT treatment. TNF-α mRNA also was increased with high glucose and reduced with A1AT (Fig 2). Protein expression values obtained for TNF-α with western blot assay are showed further in Fig 3C, where a similar expression profile is observed.

### 3.3 A1AT downregulates NFκB pathway induced under high glucose culture conditions

Transglutaminase 2 (TG2) is a protein expressed in cytoplasm and nucleus and is involved in different cellular processes like apoptosis, proliferation, cellular adhesion, motility, receptor signaling and inflammation activating NFκB pathway. This activation occurs with Akt phosphorylation. Also, TG2 inhibits a protein called PTEN, an NFκB pathway negative modulator.

In this work, we chose to evaluate some pathways targets by western blot to know how protein expression could be affected by A1AT and other targets by RT-qPCR to know mRNA expression. In our model, where cells are exposed to high glucose and A1AT treatments just for 16h, is expected than protein expression changes take more time than mRNA expression changes. During dynamic adaptation processes, the delay between transcription and translation needs to be taken into account [25]. Unless mRNA is less informative than protein expression levels, changes in mRNA expression observed may reflect changes on gene regulation due to A1AT treatment in a short time.

**Fig 4:**
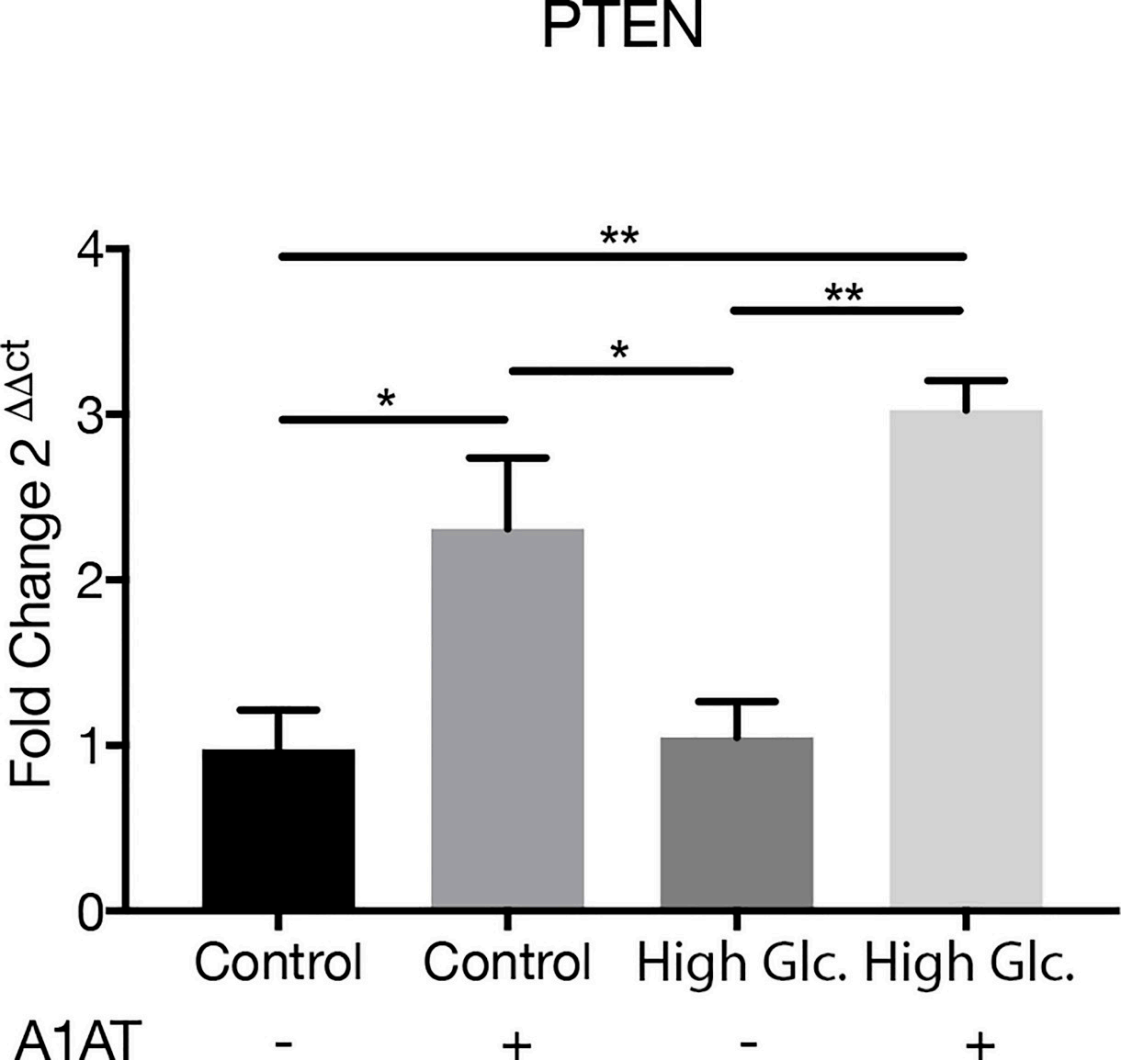
PTEN mRNA expression measured by RT-qPCR. High glucose group expressed as ‘High Glc’. Data expressed as fold change with respect to control mean ± SD. *p< 0.05, **p< 0.01 vs. Control.

Western blot (Fig 3) and RT-qPCR (Fig 4) were performed to estimate the expression of different NFκB pathway mediators under high glucose and determine how they were affected by A1AT treatment. TG2 (Fig 3A), NFκB p65 (Fig 3B) and TNF-α (Fig 3C) were overexpressed with high glucose and reduced after A1AT treatment. Particularly, a slight reduction in Akt expression is observed with A1AT treatment compared to control and high glucose (Fig 3D). This reduction was higher in the phosphorylated form of Akt (pAkt), (Fig 3E, Fig 3F). In contrast, mRNA levels of PTEN were augmented under A1AT culture conditions (Fig 4).

It is known than an augmented expression of fibroblast growth factor receptor 1 (FGFR1) promotes NFκB pathway in an inflammatory context. Also, phosphatidylinositol 3-kinase (PI3K), which also activates Akt, is a common target of FGFR1 kinases [26]. For this reason, we evaluated FGFR1 expression by western blot. As expected, an augmented expression of FGFR1 was observed in high glucose context. With A1AT treatment expression of A1AT in high glucose group was reduced but not normalized (Fig 3G).

### 3.4 A1AT modulates mediators related to glucose metabolism and epithelial-mesenchymal transition (EMT)

Akt is a key mediator in numerous pathways that involve cellular processes like cell metabolism, growth, proliferation and survival. Cellular processes related with glucose metabolism and EMT, like insulin signaling [27], mTOR [28] and Wnt canonical [29] pathways, were of particular interest to us.

#### 3.4.1 Glucose metabolism

**Fig 5:**
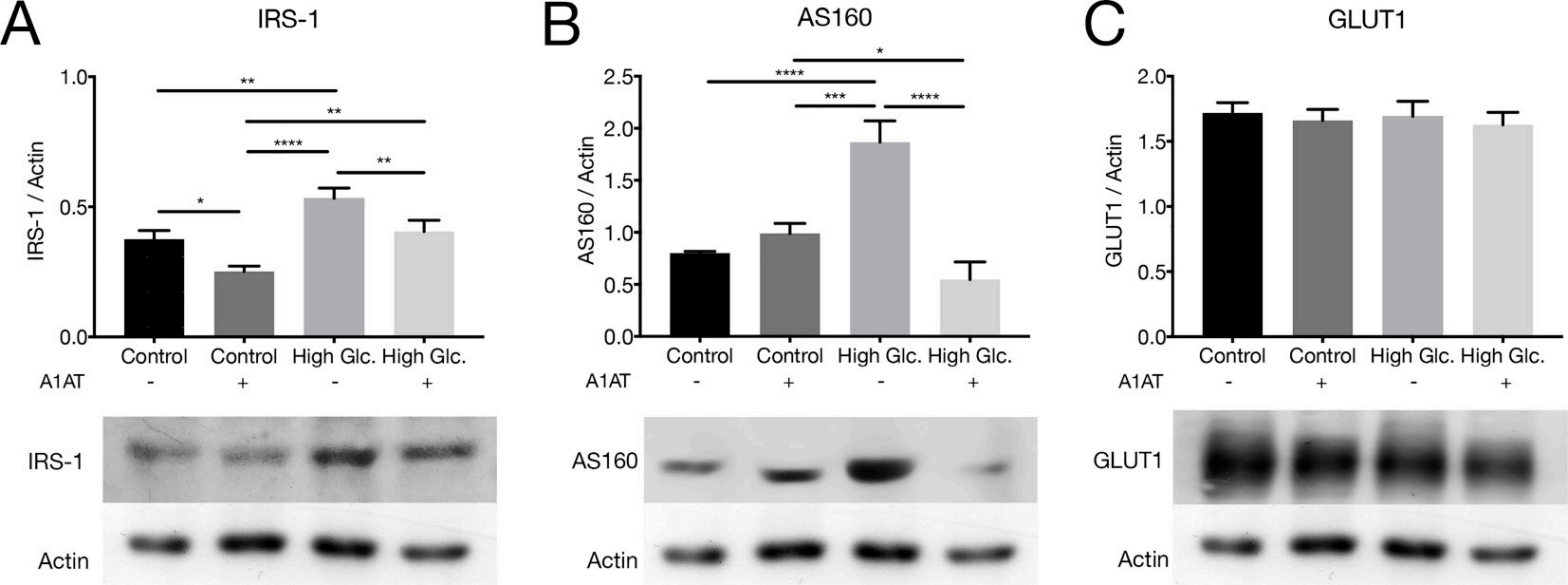
Insulin pathway proteins involved in glucose metabolism under high glucose and with A1AT treatment. Expression of different intermediates of insulin pathway was measured by western blot (A) IRS-1, (B) AS160 and (C) GLUT1. High glucose group expressed as ‘High Glc’. Results were expressed as protein vs. Actin expression mean ± SD. *p< 0.05, **p< 0.01, ***p< 0.001, ****p< 0.0001 vs. Control. Due to the different molecular weight of IRS-1, AS160, GLUT1 and Actin, the same PVDF membranes were incubated with the different antibodies, for this reason, Actin band pattern is repeated amongst the different proteins showed.

IRS-1 and AS160 are effector proteins of insulin receptor activation that finish with GLUT-1 translocation to cell membrane. At this point GLUT-1 binds glucose and transports it into the cell being available to cellular processes. IRS-1, AS160 and GLUT1 expression was measured by western blot. Overexpression was observed for IRS-1 and AS160 in high glucose groups. In both cases, these values were reduced with A1AT treatment (Fig 5A, Fig 5B). No changes on GLUT1 expression were observed amongst groups (Fig 5C).

#### 3.4.2 EMT: mTOR pathway

**Fig 6:**
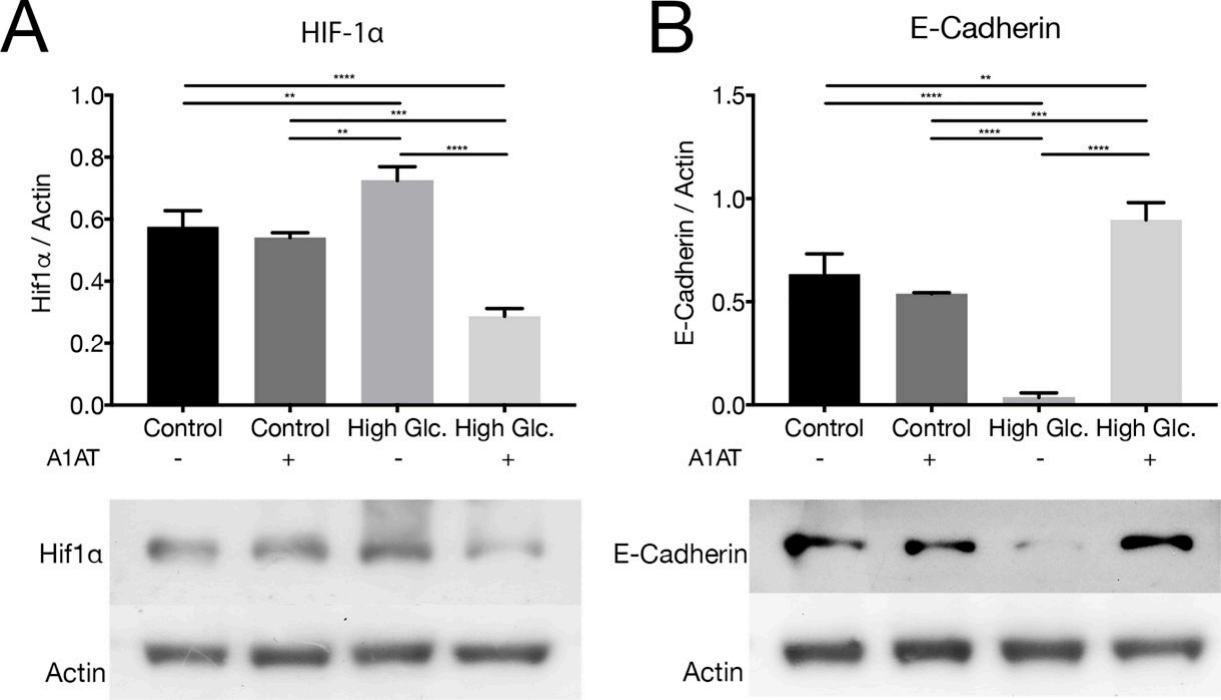
A1AT effect on mTOR and N-Cadherin mRNA. (A) RT-qPCR for MTOR mRNA, (B) RT-qPCR for CDH2 (N-Cadherin). High glucose group expressed as ‘High Glc’. Data expressed as fold change with respect to control mean ± SD.*p< 0.05, **p< 0.01, ****p< 0.0001 vs. Control.

**Fig 7:**
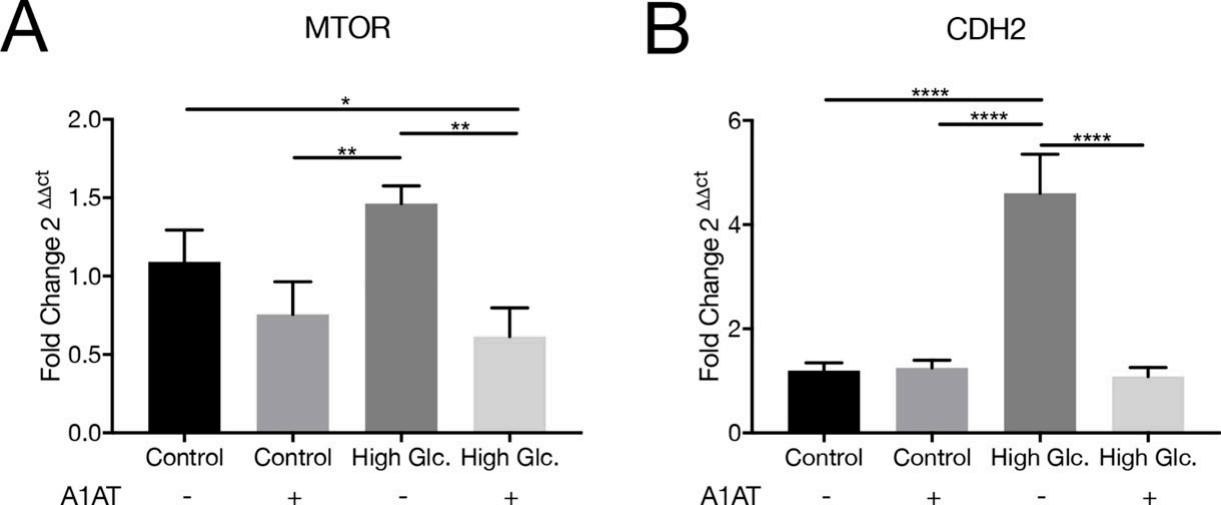
A1AT effect on mTOR pathway targets: E-Cadherin and HIF-1α. (A) HIF-1α and (B) E-Cadherin western blot assay. Results were expressed as protein vs. Actin expression mean ± SD. High glucose group expressed as ‘High Glc’. *p< 0.05, **p< 0.01, ***p< 0.001, ****p< 0.0001 vs. Control.

mTOR is a protein immediately downstream Akt in the pathway, integrating different signaling pathways like insulin and insulin growth factor. In this way, it senses oxygen, nutrients and energy levels. HIF-1α, E-Cadherin and N-Cadherin are proteins regulated with mTOR pathway, where HIF-1α is a protein related with angiogenesis, and E-Cadherin and N-Cadherin are two proteins related with cytoskeleton and cellular adhesion. RT-qPCR was performed to evaluate mTOR mRNA expression. mTOR mRNA was observed to be unmodified by high glucose concentration but was found to be downregulated with A1AT treatments (Fig 6C). HIF-1α and E-Cadherin protein expressions were evaluated as targets of Akt/mTOR pathway. HIF-1α protein expression was increased with high glucose and decreased with A1AT treatment (Fig 7A). In E-Cadherin case, the opposite pattern was observed, with a strong decrease in its expression under high glucose; however, a marked increment in presence of A1AT was observed (Fig 7B). Also, N-Cadherin (CDH2) mRNA expression was evaluated. A higher expression of this mRNA was observed in high glucose groups and restored to control features with A1AT (Fig 6B).

#### 3.4.3 EMT: Wnt canonical pathway

**Fig 8:**
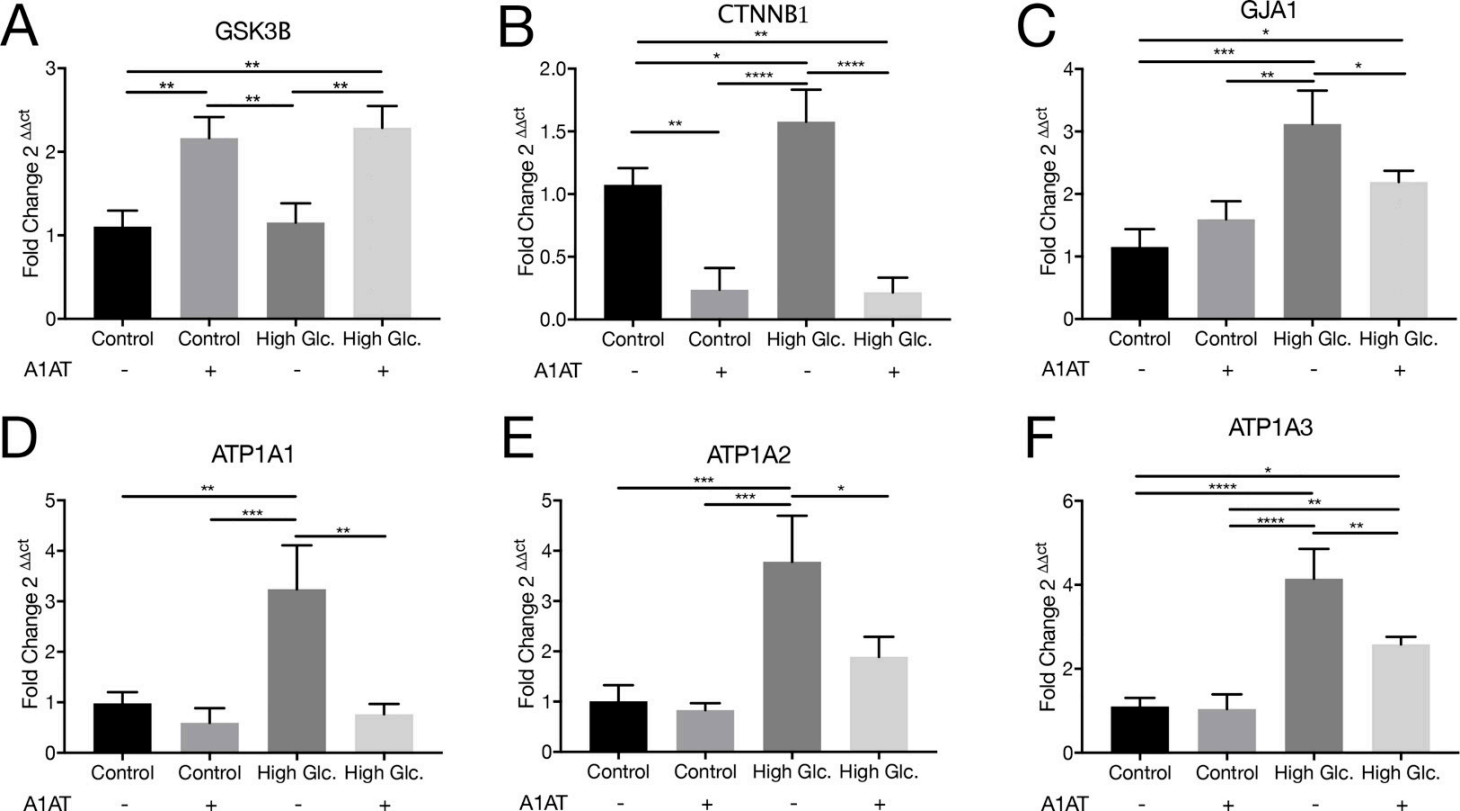
Wnt canonical pathway inhibited by A1AT. RT-qPCR was performed for Wnt canonical pathway mediators mRNA and targets regulated by this signaling pathway (A) GSK3B; (B) CTNNB1 gene related to β-Catenin expression; (C) Cx43 encoding gene GJA1; (D) ATP1A1, (E) ATP1A2, (F) ATP1A3 genes related to NKA alpha subunit isoforms α1, α2 and α3. Data expressed as fold change with respect to control. High glucose group expressed as ‘High Glc’. Results were expressed as mean ± SD. *p< 0.05, **p< 0.01, ***p< 0.001, ****p< 0.0001 vs. Control.

**Fig 9:**
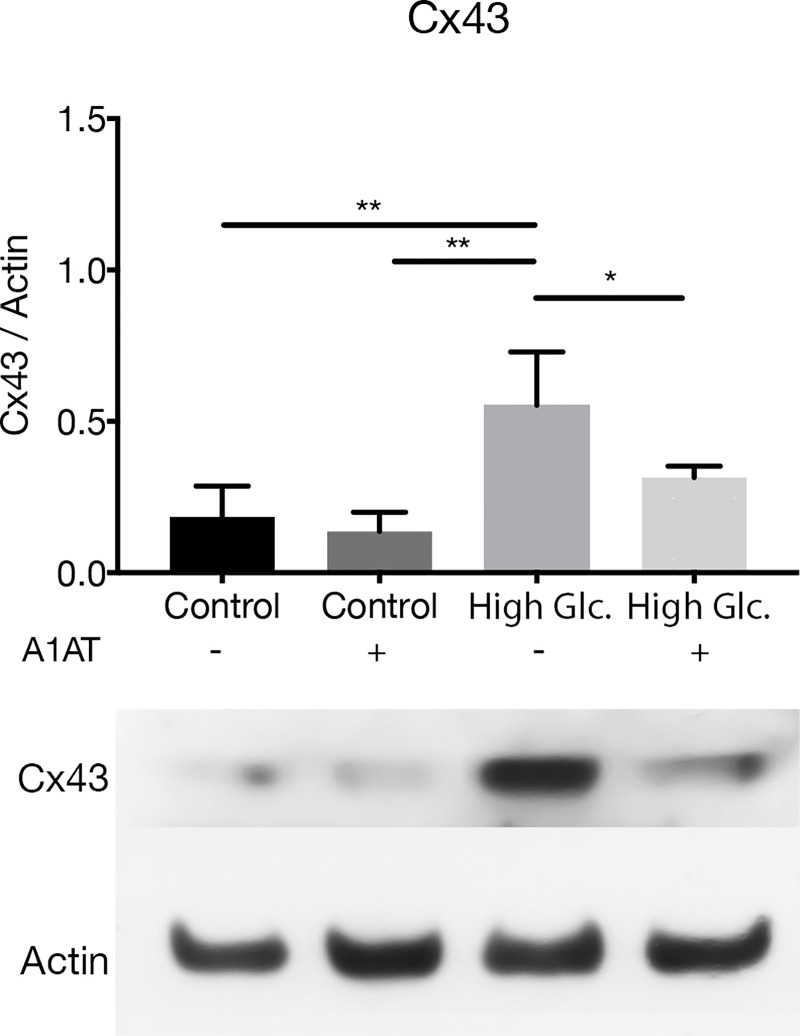
Cx43 protein expression performed by western blot. High glucose group expressed as ‘High Glc’. Results were expressed as protein vs. Actin expression mean ± SD. *p< 0.05, **p< 0.01 vs. Control.

Wnt canonical pathway is a signaling pathway involved in cell proliferation, tissue regeneration and cell migration. Wnt pathway mediators like GSK3β and β-Catenin are responsible for regulation of different proteins that are essential to cell function. This pathway is affected in diabetes and, consequently, the expression of target proteins like the alpha subunit of Na^+^/K^+^-ATPase or Cx43 is expected to be affected as well. Different components and targets of the Wnt canonical pathway were evaluated by RT-qPCR. It was shown that Akt may inhibit GSK3β, which is a repressor of β-Catenin transcription factor [30]. GSK3β mRNA expression presented an expected opposite pattern of expression compared to β-Catenin (CTNNB1) mRNA. GSK3β mRNA was upregulated with A1AT, whereas β-Catenin mRNA was decreased (Fig 8A, Fig 8B). Cx43 and the alpha subunit isoforms of Na^+^/K^+^-ATPase were selected as targets of the Wnt canonical pathway because they are essential for normal cell function [31,32]. In all cases evaluated, mRNA expression was overexpressed under high glucose concentration and decreased with A1AT treatment, consistent with β-Catenin mRNA expression (Fig 8C, Fig 8D, Fig 8E, Fig 8F). In particular, for alpha 1 and 2 isoforms of Na^+^/K^+^-ATPase pump, they were restored to control conditions (Fig 8D, Fig 8E). Finally, Cx43 was evaluated by western blot to assess if Cx43 protein expression corresponds to mRNA expression. In this case, Cx43 expression was increased under high glucose but reduced with A1AT (Fig 9).

## 4. Discussion

**Fig 10:**
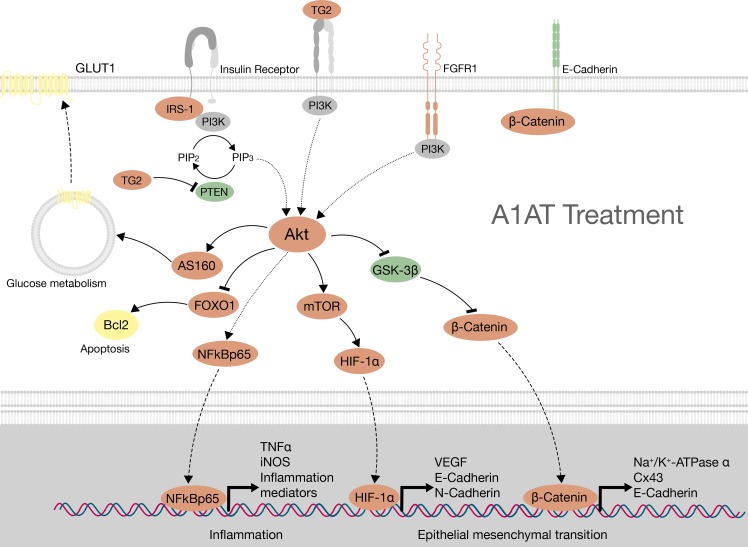
Pathways involved in A1AT treatment on ARPE-19 cells under high glucose. Red: proteins upregulated with high glucose and downregulated with A1AT treatment. Green: proteins downregulated with high glucose and upregulated with A1AT treatment. Yellow: proteins without changes in their expression.

The RPE mediates communication between neural retina and choriocapillaris, providing structural support to the retina and also contributing to its metabolism, function and integrity. During DR, where high levels of glucose are present, neurodegenerative changes occur in the retina affecting glial cells, ganglion cells, pericytes and vascular endothelial cells. RPE is also affected by hyperglycemia and its dysfunction is a contributing factor to early DR [33]. All these changes are produced before they can be detected clinically [34,35]. In turn, the retinal neurovascular unit becomes more damaged and the inner-retinal barrier is broken. Previous results from our lab have shown that A1AT treatment improves some features of the diabetic retina. Due to the relevance of RPE during DR, we tested A1AT treatment in an ARPE-19 cell culture exposed to high glucose conditions in this work. In particular, we focused on the possible pathways in which A1AT may be involved. We have found changes of mRNA and protein expression in different proteins involved in signaling pathways related to inflammation, glucose metabolism, cell survival and function (Fig 10).

First, ARPE-19 cells were tested under high glucose and A1AT culture conditions. Neither changes on cell viability nor apoptotic cells were observed. Despite FOXO1 mRNA was overexpressed with high glucose culture media, no changes in Bcl2 mRNA expression were detected. FOXO1 is a transcription factor related to cell proliferation and apoptosis, regulating the expression and activity of its downstream factors like Bcl2 [36]. However, changes in FOXO1 higher expression levels not necessarily imply an increment in Bcl2 expression or activity. Other events like phosphorylation or acetylation are needed to change Bcl2 expression and/or activity [37,38]. Also, subcellular localization is enough to affect FOXO1 targets [39]. Moreover, a different study has also found an inverse correlation in FOXO1 and Bcl2 expression [40]. All these results together indicate that ARPE-19 cells viability is not affected by the different cell culture conditions tested in this work.

NFκB is a DNA binding protein related to inflammation. This protein is overexpressed in diabetes [41] and DR pathogenesis [42] in different cell lines and animal models of diabetes. NFκB regulates the expression of pro-inflammatory molecules like TNF-α and iNOS [43]. Previous results from our lab showed that the levels of retinal TNF-α were restored to healthy values with A1AT treatment in a diabetic mouse model [18]. In the current study, we tested the NFκB, TNF-α and iNOS expression on ARPE-19 cells under high glucose and with A1AT, and obtained results that are consistent with our previous findings. As expected, a higher expression of NFκB, TNF-α and iNOS was found under high glucose culture conditions. However, these expressions were reduced using A1AT.

It is already known that the PI3K/Akt/NFκB pathway is affected in diabetes and inflammatory processes [44,45]. To understand the way the NFκB pathway was affected by A1AT treatment, intermediate proteins upstream this pathway, like TG2, FGFR1, Akt and pAkt, were evaluated. Also, PTEN mRNA expression was evaluated as a complement. TG2, FGFR1 and PTEN are proteins that activate or inhibit the PI3K/Akt pathway, respectively [46–48]. TG2 and FGFR1 are proteins activated by cytokines, like TNF-α and INF-γ, through NFκB pathway or other pathways downstream PI3K, like mTOR [26,49–51]. TG2 is also involved in EMT, triggering this process through PI3K [52]. In this work, TG2 and FGFR1 showed upregulation within the high glucose group and downregulation when A1AT was used. However, PTEN presented the opposite pattern, with a higher expression of its mRNA with A1AT treatment. In a previously published research study, PTEN was observed to be downregulated in a diabetes type 2 rat model and associated with angiogenesis. PTEN upregulation was proposed as treatment for vascular disease [50]. All these data indicate that TG2 and FGFR1 in our study are possibly overexpressed by induced pro-inflammatory stimuli. Additionally, these molecules could be activating PI3K and modulating PTEN, a fact that was previously observed in cancer [53]. All these events were corrected or improved with the use of A1AT.

Downstream in the pathway, we found the relation pAkt/Akt to be increased in RPE cells under high glucose in accordance with recent reports [54]. Surprisingly, Akt levels were downregulated by A1AT, and this effect was stronger in pAkt. The role of Akt as mediator in different pathways related not only to inflammation but also to glucose metabolism and EMT [55–57] emphasizes the importance of this result which supports the possible benefits of A1AT treatment on DR and may explain the benefits of A1AT treatment found *in vivo* [58].

The interesting results obtained for Akt with A1AT treatment prompted us to study other events associated with this protein and DR, like glucose metabolism and EMT, particularly, insulin pathway, mTOR and the Wnt canonical pathway. IRS-1 is a molecule associated with the insulin pathway, able to conduct extracellular to intracellular signals activating the PI3K/Akt pathway [59]. This protein was previously described in DR, where different patterns of phosphorylation were associated with insulin resistance and cell death [60,61]. In DR animal models, it was previously described to be augmented in different cell types like pericytes, endothelial cells and RPE cells [55,62,63]. In our study, we observed an upregulated expression of IRS-1 and its downregulation with the use of A1AT. Considering these changes observed on IRS-1, we evaluated AS160, a protein normally activated by phosphorylation by Akt and which is able to modulate GLUTs [64,65]. In ARPE-19 cells, an upregulation of AS160 was observed in our study, comparable to findings observed in type 2 diabetes studies [59,66]. This overexpression under high glucose was corrected with the use of A1AT. To find out if the changes on the expression of the evaluated proteins could have an effect on glucose uptake, we evaluated GLUT1, a glucose receptor expressed in RPE [67]. In this case, no significant changes were observed on GLUT1 expression in diabetic or A1AT treated groups. Results from reports on GLUT1 expression under high glycemia in RPE are not clear and contradictory. There are studies where GLUT1 was found to be downregulated under high glycemia [68]. On the other hand, there is a study where authors observed an upregulation of GLUT1 in similar conditions [69]. Finally, other studies mentioned no changes of GLUT1 expression, which is in line with our results [8,70].

Different types of stress, like proteotoxic stress, or high glucose produce EMT in RPE cells [71,72]. In our work, we tested different pathways involved in this process that modulate expression of proteins that are vital to this process, like Akt/mTOR, and the Wnt canonical pathway. These pathways regulate E-Cadherin and N-Cadherin expression affected in DR where Akt/GSK-3β are mediators [73]. Also mTOR signaling regulates HIF-1α expression affected as well in DR, where it is overexpressed [74]. If results obtained are taken collectively, A1AT proved to be useful for DR treatment by helping to correct E-Cadherin, N-Cadherin mRNA and HIF-1α expression. E-Cadherin levels in RPE not only have an impact on the EMT process, but also are important to maintain NKA apical polarity [75]. NKA distribution and stabilization of its localization are essential to normal function of RPE [76]. Furthermore, since NKA activity is also affected by high glucose concentrations on the RPE [77], changes on its expression or localization could affect NKA activity. We also tested NKA alpha subunit isoforms 1, 2 and 3 under high glucose and with A1AT, and found that their expression was altered under high glucose and normalized with A1AT. Likewise, β-Catenin and GSK-3β were also normalized with A1AT. Another protein regulated by the Wnt canonical pathway is Cx43. Cx43 was found to be overexpressed under high glycemia in RPE, in consonance with published data [78]. Remarkably, Cx43 was downregulated with A1AT treatment, which is consistent with findings on β-Catenin and GSK-3β and shows the benefits of A1AT treatment even for proteins related to essential processes on RPE cells.

To characterize A1AT as a simple antiserine protease seems to be an incomplete definition. Recent studies pointed out A1AT as an anti-inflammatory and immunoregulatory molecule, independent of its antiprotease activity. In our study changes in glucose metabolism, in inflammatory molecules and in Akt upstream and downstream elements were modulated by A1AT treatment.

Mechanisms behind diabetic reetinopathy are complex and involve several procesess and signaling pathways wich are interconnected. A1AT seems to be effective boarding some of the most important pathways related with them. In a few genes and proteins the comparison of control and high glucose groups treated with A1AT revealed a trend toward an opposite effect. Several factors can alter genes and proteins expressions. We might argue that some genes and proteins could be simultaneously regulated by more than one signaling pathway, condition or factor. This might be the cause of some unexpected findings. In addition, the complete mechanism of A1AT in DR is not known.

## 5. Conclusions

This research shed light on the mechanism of action of A1AT at molecular level using RPE cells, which includes impairment of inflammatory events, regulation of proteins related to glucose metabolism, awakening of signals related to EMT and normalization of protein levels involved in essential RPE function. So, in addition to anti-protease effects A1AT seems to have other positive outcomes on RPE cells exposed to high glucose. Further research is needed to support all molecular mechanisms involved in A1AT treatment. Yet, these findings contribute to a better understanding of the benefits of using A1AT as a new treatment for DR and its potential for use in other retinal diseases that share the underlying mechanisms.
